# The prognostic value of Presepsin for postoperative complications following pancreatic resection: A prospective study

**DOI:** 10.1371/journal.pone.0243510

**Published:** 2020-12-09

**Authors:** Silvia Gasteiger, Florian Primavesi, Peter Werkl, Lucie Dostal, Philipp Gehwolf, Eva Braunwarth, Manuel Maglione, Sieghart Sopper, Dietmar Öfner, Stefan Stättner

**Affiliations:** 1 Department of Visceral, Transplant and Thoracic Surgery, Medical University of Innsbruck, Innsbruck, Austria; 2 Department of Surgery, Salzkammergut Klinikum, Vöcklabruck, Austria; 3 Department of Medical Statistics, Informatics and Health Economics, Medical University of Innsbruck, Innsbruck, Austria; 4 Department of Haematology and Oncology, Medical University of Innsbruck, Innsbruck, Austria; UKSH Campus Lübeck, GERMANY

## Abstract

**Background:**

Presepsin is involved in binding lipopolysaccharides and previous studies have confirmed its value as a marker for early diagnosis and prediction of severity in sepsis. Comparable studies assessing the predictive potential regarding postoperative complications and mortality following pancreatic resection are lacking.

**Methods:**

This prospective study included 70 patients undergoing pancreatic resection from December 2017 until May 2019. Presepsin was measured preoperatively, on postoperative day 1, 3 and 8 (POD1/3/8) and correlated with the clinical course and mortality.

**Results:**

Severe complications (Clavien-Dindo ≥3a) occurred in 28 patients (40%), postoperative pancreatic fistula (POPF) grade B/C occurred in 20 patients (28.6%), infectious complications in 28 (40%), and four patients (5.7%) died during hospital stay. Presepsin levels at any timepoint did not correlate with further development of postoperative complications or in-hospital mortality whereas CRP levels on postoperative day (POD) 3 were significantly associated with clinically relevant POPF (AUC 0.664, 95%CI 0.528–0.800; p = 0.033). Preoperative Presepsin levels as well as Presepsin on POD1 were significantly elevated in patients with malignant compared to benign underlying disease (299pg/ml vs. 174pg/ml and 693.5pg/ml vs. 294pg/ml; p = 0.009 and 0.013, respectively).

**Conclusion:**

In our cohort, Presepsin was not eligible to predict the postoperative course following pancreatic resection. However, Presepsin levels were significantly elevated in patients with malignant disease, this finding warrants further investigation.

## Introduction

Pancreatic resections (PR) are associated with significant morbidity and mortality. Despite improvements in surgical technique, as well as peri- and postoperative care, mortality rates nowadays range between 2 and 5%, in most centres [1, 2]. Moreover, morbidity rates after PR can be as high as 70%, especially when evaluated in a prospective setting [1, 3]. Postoperative pancreatic fistula (POPF) represents one of the most frequent complications following PR, with a high variability in the literature ranging from 20% to 64% [4–6]. Other common complications include delayed gastric emptying (DGE), postoperative haemorrhage (PPH), or intra-abdominal fluid collections [7–9]. All of these complications are often accompanied by underlying infection, and in case of delayed detection or insufficient therapy are at high risk of potential sepsis and mortality [10, 11].

The most commonly used biomarkers to detect postoperative complications are C-reactive protein (CRP), white blood cell count (WBC), and procalcitonin (PCT). However, all these biomarkers have the disadvantage that they may show elevated levels due to various causes including infection, inflammation and surgical trauma and thus, are often not precise enough for an exact clinical assessment. Presepsin (sCD14-ST) is a novel biomarker and has recently been investigated for the prediction of infectious complications by various medical disciplines with promising results [12, 13]. Presepsin is a fragment of the glycoprotein CD14, which can be found on monocytes, macrophages, and neutrophil granulocytes. CD14 is a receptor triggered by the lipopolysaccharides (LPS)—lipopolysaccharide binding protein (LPB) complex (CD14-LPS-LBS) [14]. The subsequent activation of TLR4 (toll like receptor) further conducts the release of proinflammatory cytokines, which leads to a systemic inflammatory response [15, 16]. Then, CD14-LPS-LBS gets internalized into a phagolysosome. Finally, its soluble fraction (sCD14) gets cleaved in the bloodstream by proteases into 64 amino acid long peptides. This sCD14 subtype (sCD14-ST) commonly called Presepsin [16, 17] can be detected in whole blood and plasma with point-of-care testing [18].

Studies investigating the value of Presepsin predicting morbidity following major HPB procedures are lacking. Accordingly, the aim of the present study was to evaluate the perioperative dynamics of Presepsin and its predictive potential as a marker of postoperative complications following PR compared to other markers already established in clinical routine.

## Materials and methods

The present study was approved by the local ethics committee of Medical University of Innsbruck (study number 1081/2017). All patients undergoing distal pancreatectomy or pancreatic head resection at the Department of Visceral, Transplant and Thoracic Surgery, Medical University of Innsbruck, Austria between December 2017 and May 2019 were included. Cases with unresectability detected during surgical exploration were excluded. Written informed consent was obtained from all patients before enrolment. The present study was registered at clinicaltrials.gov (NCT04294797). It was conducted and the results reported in accordance with the 2013 Helsinki World Medical Association Declaration (PMID: 24141714) and the STROBE checklist [19].

In addition to routine laboratory parameters (WBC count, CRP, procalcitonin, kidney and liver function parameters, amylase and lipase), systemic Presepsin values were assessed preoperatively and on POD 1, POD 3 and POD 8 following PR. Postoperative morbidity and mortality were recorded prospectively during hospital stay through our surgical units’ auditable electronic database (ChiBase). Complications were graded according to the Clavien-Dindo classification of surgical complications [20–22]. POPF was classified as defined in the 2016 update of the International Study Group for Pancreatic Surgery (ISGPS) grading of postoperative pancreatic fistula [23]. Postpancreatectomy haemorrhage (PPH) and delayed gastric emptying (DGE) were defined according to the respective 2007 definitions of the ISGPS [24, 25].

### Test device and test kits

The test device Pathfast Compact immuno-analyser^™^ was provided by the company Axon Lab AG (Polling, Austria). The Pathfast^™^ is a multi-analysis instrument capable to perform in vitro quantitative analysis of different components of whole blood, heparinized plasma and serum. The test kits including the reagents used for the Pathfast immuno-analyser^™^ are manufactured by LSI Medience Corporation. The measurement principle is based on CLEIA (chemiluminescence enzyme immunoassay). Whole blood Presepsin levels were measured according to the manufacturers’ protocol.

Measurement and documentation of the obtained Presepsin values was accomplished by the study coordination office to avoid a possible influence on patient therapies by the treating physicians, who were blinded to the test results.

### Statistical analysis

Data are reported as median (range) or numbers with percentages. Baseline characteristics and Presepsin values as predictors for major complications or in-hospital mortality were analysed using Mann Whitney U test, Chi-square test or Wilcoxon test, as appropriate. ROC-analysis was performed to assess Presepsin regarding association with severe complications (Clavien-Dindo ≥3a), POPF grade B/C, infectious complications, DGE, PPH and in-hospital mortality. All p-values were based on a 2-sided test and p-values <0.05 were considered statistically significant. Data was analysed using IBM SPSS Statistics version 23 (IBM, Armonk, New York, USA).

## Results

### Patient characteristics

A total of 70 patients were included in the present study, with 45 cases (64.3%) undergoing pancreatic head resection and 25 cases (35.7%) receiving distal pancreatectomy (16/25 laparoscopically). Patient characteristics and surgical details are displayed in Table 1. The indication for PR was malignant disease in 56 (80%) of patients. Median age at time of surgery was 64.5 years (range 25–88) and 32 (45.7%) were female.

**Table 1:** Patient characteristics and surgical details (n = 70).

**Patient Characteristics**	70 (100%)
Age; median (range)	64.5 (25–88)
Female sex	32 (45.7)
BMI; median (range)	25.1 (16.1–43)
Chronic pancreatitis	8 (11.4)
Pre-existing diabetes mellitus	12 (17.1)
**Surgical procedure**	
PPPD	45 (64.3)
Laparoscopic distal pancreatectomy	16 (22.9)
Open distal pancreatectomy	9 (12.9)
**Indication**	
PDAC	36 (51.4)
pNET	12 (17.1)
dCCA	4 (5.7)
IPMN	4 (5.7)
PanIN	1 (1.4)
Chronic pancreatitis	3 (4.3)
Other	10 (14.3)

BMI, body mass index; PPPD, pylorus-preserving pancreatoduodenectomy; PDAC, pancreatic ductal adenocarcinoma; NET, neuroendocrine tumour; dCCA, distal cholangiocellular carcinoma; IPMN, intraductal papillary mucinous neoplasm; PanIN, intraepithelial neoplasm.

### Morbidity and mortality

Median length of hospital stay (LOS) was 13 days (range 5–64). Postoperative complications including minor complications (Clavien-Dindo 1 & 2) occurred in 53 patients (75.7%). Severe complications occurred in 28 patients (40%). Clinically relevant POPF grade B/C (CR-POPF) occurred in 20 patients (28.6%) including 13 (18.6%) POPF grade B and 7 (10%) POPF grade C. Eight (11.4%) patients suffered from DGE and 7 (10%) from PPH, infectious complications occurred in 26 patients (37.1%). Four patients (5.7%) died during hospital stay, all of them suffering from malignant disease. Cause of in-hospital death was cardiac infarction in 2 patients, and surgery related in another two patients: multiorgan failure (MOF) due to gastric ischemia following distal pancreatectomy with concomitant partial gastric resection in one patient and MOF due to insufficiency of pancreatojejunostomy and hepaticojejunostomy in another patient, resulting in a failure to rescue rate of 7.5%.

### Perioperative dynamics of Presepsin levels

**Fig 1:**
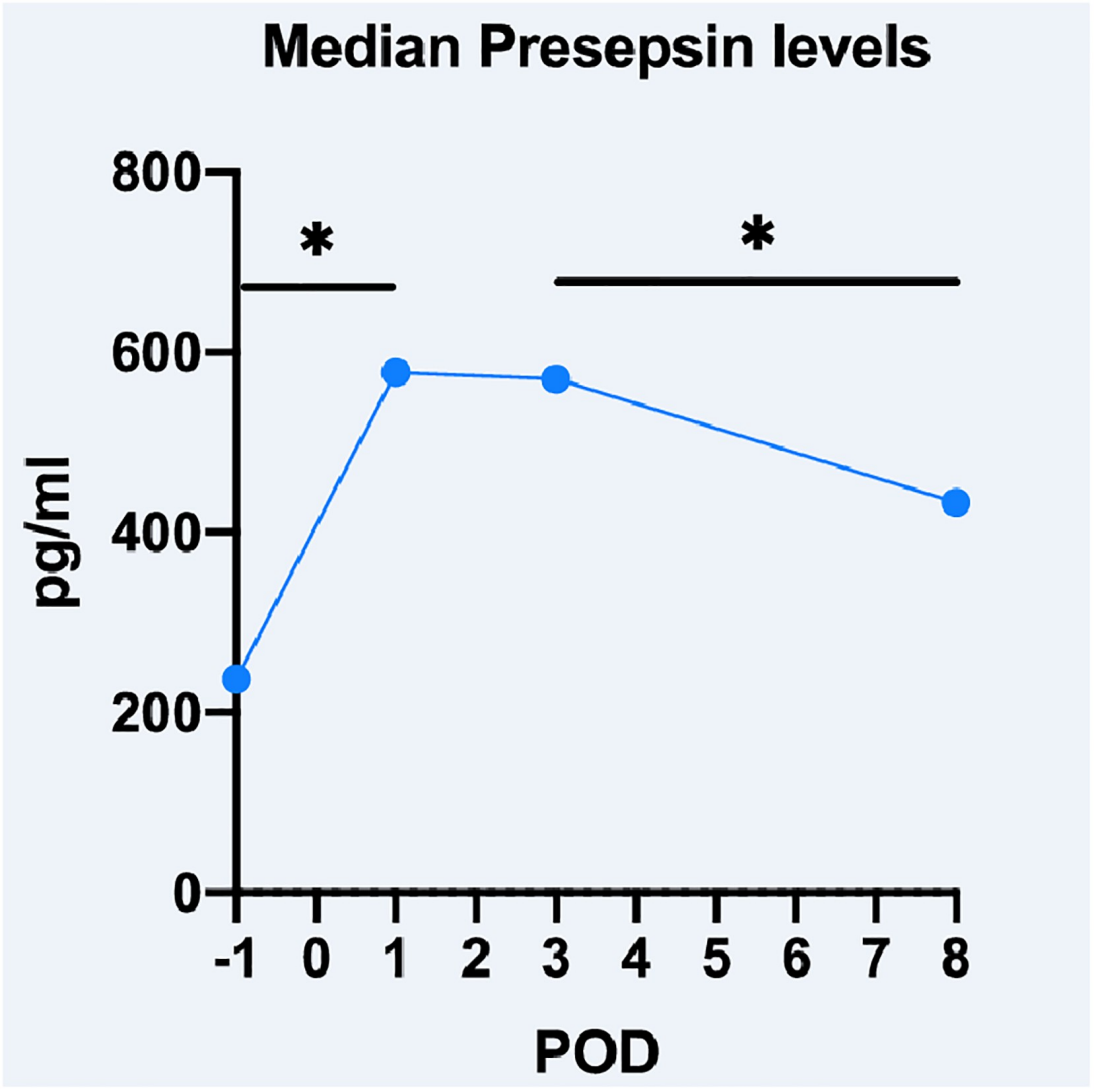
Perioperative dynamics of Presepsin.

The median preoperative Presepsin value was 237pg/ml (range 102–7,375pg/ml; Fig 1). Presepsin values increased significantly on POD1 with a median value of 577.5pg/ml (range 133–7,386pg/ml, p<0.001). Median Presepsin level at POD3 was 570.5pg/ml (range 197-7253pg/ml) and declined thereafter significantly to 433pg/ml (range 112pg/ml-4640pg/dl, p<0.001).

Presepsin values did not differ among gender, age (<65 years vs. ≥65 years) or surgical approach (laparoscopic vs. open surgery) at any timepoint (Table 2). However, preoperative and POD1 values where significantly lower in patients with benign compared to malignant underlying disease (174pg/ml vs. 299pg/ml and 294pg/ml vs. 693.5pg/ml; p = 0.013 and 0.009, respectively).

**Table 2:** Presepsin values stratified by gender, age, indication for PR and surgical approach.

Presepsin values	Preoperative	p-value	POD1	p-value	POD3	p-value	POD8	p-value
**Overall cohort**	237 (102–7,375)		577.5 (133–7,386)		570.5 (197–7,253)		433 (112–4,640)	
**Gender**		0.511		0.067		0.850		0.635
Male	221 (102–7,375)		666 (133–7,386)		614 (197–7,253)		444 (173–4,640)	
Female	262 (126–4,422)		444 (142–7,350)		560.5 (201–6,086)		427 (112–2,556)	
**Age**		0.644		0.698		0.125		0.750
<65	194 (108–7,375)		524 (142–7,386)		471 (201–7,253)		404.5 (139–4,640)	
≥65	285 (102–4,422)		604 (133–7,350)		675 (197–6,086)		455.5 (112–2,556)	
**Indication**		**0.013**		**0.009**		0.533		0.062
benign	174 (108–609)		294 (205–1,348)		533 (245–987)		271 (175–2,556)	
malign	299 (102–7,375)		693.5 (133–7,386)		591.5 (197–7,253)		448 (112–4,640)	
**Surgical approach**		0.512		0.713		0.559		0.926
open	171 (128–306)		408 (142–1,103)		607 (245–2,267)		303 (112–1,353)	
laparoscopic	156 (108–968)		295 (133–1,414)		483 (201–807)		327 (139–807)	

POD, postoperative day. Values are median (range.)

### Association with clinical outcome parameters

Table 3 shows ROC analysis of Presepsin, CRP, WBC count and procalcitonin and their predictive value for development of postoperative complications Clavien-Dindo≥3a, pancreas specific complications (CR-POPF, DGE and PPH), in-hospital mortality and infectious complications.

**Table 3:** Presepsin, CRP, WBC count and procalcitonin and their predictive value for development of complications Clavien-Dindo ≥3a, POPF grade B/C, DGE, PPH, in-hospital mortality and infectious complications.

	Presepsin		CRP		WBC count		Procalcitonin	
Complications C-D ≥3a	AUC (95%CI)	p-value	AUC (95%CI)	p-value	AUC (95%CI)	p-value	AUC (95%CI)	p-value
Preoperative	0.424 (0.289–0.599)	0.285	0.577 (0.440–0.713)	0.281	0.507 (0.366–0.647)	0.924	0.430 (0.286–0.574)	0.345
POD1	0.547 (0.411–0.682)	0.510	0.628 (0.495–0.762)	0.070	0.489 (0.354–0.624)	0.876	0.564 (0.424–0.703)	0.372
POD3	0.583 (0.444–0.722)	0.242	0.611 (0.477–0.744)	0.119	0.528 (0.386–0.670)	0.697	0.616 (0.473–0.760)	0.121
POD8	0.613 (0.475–0.752)	0.114	0.784 (0.669–0.898)	**<0.001**	0.756 (0.635–0.877)	**<0.001**	0.691 (0.551–0.830)	**0.010**
**POPF grade B/C**								
Preoperative	0.402 (0.258–0.545)	0.209	0.472 (0.325–0.618)	0.711	0.620 (0.475–0.765)	0.119	0.397 (0.246–0.547)	0.200
POD1	0.393 (0.252–0.534)	0.164	0.514 (0.355–0.673)	0.856	0.612 (0.481–0.743)	0.145	0.477 (0.305–0.650)	0.773
POD3	0.506(0.357–0.654)	0.943	0.664 (0.528–0.800)	**0.033**	0.622 (0.467–0.777)	0.113	0.531 (0.343–0.709)	0.704
POD8	0.478 (0.333–0.623)	0.779	0.774 (0.667–0.882)	**<0.001**	0.718 (0.585–0.851)	**0.006**	0.634 (0.478–0.789)	0.099
**DGE**								
Preoperative	0.393 (0.180–0.607)	0.330	0.283 (0.111–0.456)	0.074	0.451 (0.232–0.669)	0.651	0.386 (0.149–0.622)	0.359
POD1	0.514 (0.320–0.708)	0.897	0.649 (0.480–0.818)	0.172	0.401 (0.244–0.558)	0.366	0.772 (0.647–0.896)	**0.013**
POD3	0.514 (0.294–0.734)	0.897	0.615 (0.428–0.802)	0.293	0.319 (0.118–0.519)	0.097	0.694 (0.530–0.858)	0.096
POD8	0.575 (0.347–0.803)	0.493	0.625 (0.443–0.807)	0.253	0.640 (0.401–0.879)	0.202	0.760 (0.643–0.877)	**0.018**
**PPH**								
Preoperative	0.395 (0.172–0.618)	0.366	0.528 (0.307–0.750)	0.807	0.390 (0.189–0.591)	0.342	0.400 (0.206–0.595)	0.391
POD1	0.625 (0.415–0.835)	0.282	0.570 (0.303–0.838)	0.544	0.454 (0.226–0.681)	0.688	0.589 (0.380.0.797)	0.444
POD3	0.574 (0.312–0.835)	0.525	0.576 (0.370–0.782)	0.512	0.460 (0.232–0.688)	0.732	0.665 (0.485–0.845)	0.186
POD8	0.637 (0.402–0.872)	0.238	0.614 (0.421–0.806)	0.328	0.598 (0.354–0.843)	0.397	0.574 (0.301–0.847)	0.526
**In-hospital mortality**								
Preoperative	0.483 (0.281–0.684)	0.991	0.619 (0.267–0.971)	0.425	0.735 (0.595–0.874)	0.117	0.282 (0.077–0.487)	0.205
POD1	0.557 (0.386–0.728)	0.704	0.769 (0.574–0.964)	0.072	0.606 (0.450–0.762)	0.479	0.473 (0.183–0.763)	0.857
POD3	0.629 (0.251–1.000)	0.390	0.708 (0.437–0.979)	0.164	0.598 (0.279–0.918)	0.511	0.653 (0.372–0.933)	0.310
POD8	0.582 (0.254–0.910)	0.584	0.789 (0.598–0.980)	0.054	0.906 (0.817–0.995)	**0.007**	0.733 (0.491–0.975)	0.121
**Infectious complications**								
Preoperative	0.485 (0.344–0.626)	0.837	0.535 (0.396–0.647)	0.627	0.560 (0.422–0.699)	0.269	0.446 (0.299–0.593)	0.471
POD1	0.408 (0.273–0.544)	0.202	0.530 (0.387–0.673)	0.675	0.505 (0.368–0.641)	0.947	0.466 (0.312–0.620)	0.644
POD3	0.487 (0.346–0.629)	0.072	0.513 (0.372–0.654)	0.855	0.504 (0.359–0.649)	0.956	0.487 (0.324–0.649)	0.860
POD8	0.591 (0.447–0.734)	0.215	0.817 (0.713–0.921)	**<0.001**	0.657 (0.516–0.798)	**0.032**	0.731 (0.593–0.870)	**0.003**

C-D, Clavien-Dindo; POD, postoperative day.

There was no association of Presepsin levels with postoperative complications or in-hospital mortality at any timepoint. On POD3, CRP levels correlated with further development of POPF grade B/C. On POD8, CRP, WBC count and procalcitonin were associated with postoperative complications Clavien-Dindo ≥3a. CRP and WBC count on POD8 did also correlate with POPF grade B/C. Procalcitonin levels on POD1 and POD8 were associated with DGE. On POD8, WBC count was significantly associated with in-hospital mortality whereas CRP levels did just not reach statistical significance. On POD8 WBC count, CRP and PCT were significantly associated with infectious complications.

## Discussion

Novel markers for early detection of patients at risk for major postoperative complications and mortality could guide clinical interventions on time and thus be decisive for the patient’s prognosis. Therefore, the aim of the present study was to evaluate Presepsin (sCD14-ST) in predicting the postoperative course following pancreatic resection.

In our cohort, Presepsin levels did not correlate with further development of postoperative complications or in-hospital mortality. Early postoperative (until POD3), only CRP was associated with clinically relevant POPF grade B/C (AUC 0.664, 95%CI 0.528–0.800; p = 0.033). Nonetheless, a diagnostic test is considered acceptable if its AUC is ≥ 0.8 [26]. In our population, only CRP on POD8 and WBC count on POD8 reached this cut-off for predicting infectious complications and in-hospital mortality, respectively.

This data is in contrast to prior published data of several different surgical disciplines: Marazzi et al. assessed Presepsin as a potential biomarker for periprostetic joint infection in a cohort of 30 patients with periprostetic joint infection compared to 30 without. In their series, Presepsin showed greater diagnostic value as assessed by ROC-AUC than established markers for postoperative infections such as CRP or IL-6, namely AUC 0.926 compared to AUC 0.750 and AUC 0.821 [27]. Also in cardiac surgery patients, Presepsin has shown a comparable predictive value for major adverse events as procalcitonin and even a better predictive value for in-hospital and 6-months mortality [28]. Recently, Presepsin has also been evaluated in patients undergoing emergency visceral surgery for abdominal infection. Bösch et al. found in their small series of 31 patients Presepsin levels on the day of emergency surgery as a valuable marker for stratifying the sepsis risk and ROC analysis showed the highest AUC (0.864, 95%CI: 0.733–0.995) for predicting 90-day mortality for Presepsin as compared to other common markers for infection [29].

There might be several explanations behind this discrepancy between our data and prior published studies. First of all, the time point (postoperative day) of specimen measurement could be a relevant factor. Pancreas specific complications, especially those from insufficiently drained fluid collections usually become clinically significant several days after surgery when secondary complications such as bacterial superinfection or disastrous events such as haemorrhage occur. This would be in line with our findings that on POD8, CRP and WBC count were significantly associated with major postoperative complications and POPF grade B/C. On the other hand, WBCs are the main effector cells the immune system and their elevation could just be the response to the surgical trauma and not to an infection. Similarly, CRP as an acute phase protein can be increased in various types of systemic inflammation including infectious and non-infectious. In contrast, Presepsin is generated by the phagocytosis of bacteria [17], thus seems to be specific to bacterial infection. This would be in line with the recently reported data of Kang et al. [30] They compared Presepsin levels and other established markers of infections among infected trauma patients, non-infected trauma patients and patients undergoing sterile surgery. Presepsin levels were elevated only in infected trauma patients whereas CRP, WBC count and PCT were elevated in infected and non-infected trauma patients [30]. Nonetheless, Presepsin levels in our patient cohort did not correlate with any postoperative adverse event, whereas at least on POD8 the other mentioned markers were associated with major postoperative complications and infectious. Thus, our working hypothesis of complications following PR mostly emerging due to concomitant infection, could not be confirmed.

Lastly, in our cohort, Presepsin levels did not differ among gender, age or surgical approach, but patients with malignant disease had higher preoperative and POD1 levels compared to patients with benign indication for PR. To our knowledge, this has not been described so far, but several clinical and epidemiological studies have already demonstrated, that elevated plasma inflammatory cytokines are associated with significant reduction in cancer-related patient survival [31]. This tumour-associated inflammatory conditions in cancer patients could be a possible explanation but needs further investigation in a larger population. On the other hand, the pancreas is not a sterile organ and 1000-fold increase of intrapancreatic bacteria [32, 33] as well as fungal colonization was demonstrated in PDAC tissue compared to healthy pancreatic tissue [34]. For example, Proteobacteria, Synergistetes and Euryarchaeota are significantly more abundant in PDAC patients. While Proteobacteria comprise only 8% of gut microbiota, this species constitutes up to 50% in PDAC tissue [32]. Moreover, several oral microbes have shown correlation with PDAC: Fusobacterium spp. were independently associated with worse prognosis in PDAC patients. Also, Porphyromonas gingivalis may be associated with pancreatic carcinogenesis, while high levels of antibodies against P. gingivalis correlate with a lower risk of pancreatic cancer [35]. Since our current knowledge suggests, that elevated Presepsin levels are specific for bacterial infections, this abundance of microbiota in PDAC patients could be another explanation for the increased Presepsin levels in cancer patients.

To conclude, in our patient collective, Presepsin levels did not correlate with postoperative complications or in-hospital mortality. Early postoperative (until POD3) only CRP was associated with clinically relevant POPF. Moreover, we found higher preoperative and POD1 Presepsin levels in patients with malignant disease, this finding needs to be further investigated.

## Abbreviations

BMI: body mass index
CRP: C-reactive protein
CR-POPF: clinically relevant postoperative pancreatic fistula
dCCA: distal cholangiocellular carcinoma
DGE: delayed gastric emptying
HPB: hepato-pancreatico-biliary
IPMN: intraductal papillary mucinous neoplasm
ISGPS: international study group for pancreatic surgery
LOS: length of hospital stay
LPB: lipopolysaccharide binding protein
LPS: lipopolysaccharides
MOF: multi organ failure
NET: neuroendocrine tumour
PanIN: pancreatic intraepithelial neoplasia
PCT: Procalcitonin
PDAC: pancreatic ductal adenocarcinoma
POD: postoperative day
POPF: postoperative pancreatic fistula
PPH: postoperative pancreatic haemorrhage
PPPD: pylorus-preserving pancreatic head resection
PR: pancreatic resection
TLR: toll like receptor
WBC: white blood cell
